# *Parkinson's Elevated*: improving healthspan

**DOI:** 10.3389/fspor.2025.1529075

**Published:** 2025-06-02

**Authors:** Kathleen E. McKee, Miriam R. Rafferty, Theadora W. Sakata, David M. Hedges, Garett J. Griffith, Maddison M. K. Bingham, Stephanie A. Obradovich, M. Nicholas Francis, Daniel M. Corcos

**Affiliations:** ^1^ Department of Neurology, Intermountain Medical Center Neurosciences Institute, Intermountain Health, Salt Lake City, UT, United States; ^2^ Shirley Ryan AbilityLab, Chicago, IL, United States; ^3^ Department Physical Medicine & Rehabilitation, Northwestern University, Chicago, IL, United States; ^4^ Department of Family Medicine, Intermountain Health, Salt Lake City, UT, United States; ^5^ Healthcare Delivery Institute, Intermountain Health, Salt Lake City, UT, United States; ^6^ Department of Analytics, Select Health, Salt Lake City, UT, United States; ^7^ Department of Physical Therapy and Human Movement Sciences, Northwestern University, Chicago, IL, United States; ^8^ Neurologic Physical Therapy, Intermountain Park City Hospital, Park City, UT, United States; ^9^ Lifestyle Medicine and Wellness Center, Intermountain Park City Hospital, Park City, UT, United States

**Keywords:** Parkinson's disease, healthspan, integrated practice unit, proactive care, high-intensity aerobic exercise

## Abstract

As Parkinson's disease (PD) progresses, relatively mild symptoms advance to a major disorder that affects every organ system in the body. Current care for people with PD (PwP) reacts to rising disability. There is a missed opportunity to keep PwP as healthy as possible. In this perspective, we spell out our vision for a proactive, value-based health care model built around a patient-centered integrated practice unit (IPU) for PD. The IPU will provide integrated interdisciplinary care overseen by a specialized Parkinson's primary care physician working closely with a movement disorders neurologist. The IPU will implement an evidence-based exercise program for people early in the disease. The focus of this intervention is a heart rate driven high-intensity aerobic exercise program, which is the only treatment with evidence that it can slow disease progression. It will also include resistance exercises, flexibility exercise and balance exercise. For people whose disease is moderate or severe, the IPU will provide care curated through a network of rehabilitation providers with expertise in PD all of whom understand the exercise prescription. By integrating care, slowing disease progression, and incorporating specialized rehabilitation we anticipate improving healthspan. In creating the IPU as a fully capitated (shared-risk) model in which the IPU and the insurance company assume joint accountability for quality and cost of care we anticipate demonstrating financial sustainability of implementing the exercise prescription and providing integrated care.

## 1 Introduction

The dominant narrative of Parkinson's disease (PD) in the United States is one of rising disability managed reactively through a fragmented healthcare system (1). The first goal of this perspective is to propose a new narrative: one that keeps people with PD (PwP) healthy. This is accomplished through an integrated proactive model that extends healthspan. Healthspan is defined as the period of life spent with relatively good physical and mental function (2). The second goal of this perspective is to make clear that financial incentives must be realigned to support what is best for the patient, not what is best for the health system, physician, or insurance company. Without such realignment the new model of care we propose will never be financially viable.

We suggest an Integrated Practice Unit (IPU) (3, 4) as a vehicle through which financial incentives can be harmoniously aligned with best clinical practice. In an IPU the health providers and the insurance company share joint accountability for quality and cost of care. Integrated care is already a suggested best practice in PD (1, 5, 6) and there are centers across the United States providing at least partially integrated services. These include The Struthers Parkinson's Center in Minnesota and the Neuromedicine Service and Science Hub Model at the University of Florida (7). Parkinson's Foundation Centers of Excellence also provide more comprehensive care than most clinics. Yet, none of these centers exist as Integrated Practice Units in the true definition of the term because none have assumed joint accountability for quality *and* cost of health care (8). Through examples from our ongoing efforts to implement the *Parkinson's Elevated* IPU at Intermountain Health in Salt Lake City, UT we highlight real-world barriers and solutions to completely reshaping the narrative of PD from one of disability to health.

## 2 Current problems in the Parkinson's journey

Clinical care for PD unfolds under a chronic illness model in which the patient-physician dyad reacts to symptoms as they arise (9). We worked with our Parkinson's Patient Family Advisory Committee (PFAC) and reviewed the literature to better understand the current state of PD care in the United States. Our patients and their carers shared that many PD journeys start with a diagnostic odyssey in which the patient sees various specialists (e.g.,: orthopedist for ‘frozen shoulder,’ gastroenterologist for severe constipation, primary care for tremor) before enough motor symptoms manifest to raise the possibility of parkinsonism as a root cause for all symptoms. The subsequent delivery of the diagnosis is notoriously poor; one member of our PFAC shared that after her abrupt diagnosis, she was left so unsupported by her care team that she lost two years in unfounded despair because she thought her life was over. Her experience has been echoed in published reports (10, 11).

In those initial years after diagnosis, patients are prescribed dopamine replacement therapy and managed at ∼3–6-month intervals by neurologists. Review of the literature reveals that these patients are lucky to even see a neurologist. Wait times for neurologic care are high and according to Medicare claims data one-third of people with PD do not receive any regular neurologic care (12). PwP are generally sent to physical therapy (PT) only if they have impaired balance or gait, with utilization increasing only as the disease progresses (13). They might be told verbally that exercise is important for PD, but little is done to reinforce or support this. Referrals to sub-specialists such as sleep medicine, gastroenterology, and urology may be placed to address non-motor symptoms of the disease, but patients are left on their own to navigate between these providers (11). Patients are also expected to coordinate their care between their neurologist and their primary care physician (PCP).

It is only when disability increases and patients start falling and aspirating that services escalate: more referrals to PT/occupational therapy/speech-language pathology, more frequent primary care and neurology visits (13). Inevitably, an event such as pneumonia or hip fracture necessitates inpatient admission; the literature confirms PwP are more likely to have unplanned hospital admissions than their age-matched peers (14). From there, PwP are less likely to return to their pre-morbid place of residence and have higher in-hospital mortality than their age matched peers (15). Finally, when PwP transition to hospice they usually have no further contact with their movement physician and PCP as per hospice regulations.

## 3 The ideal Parkinson’s journey

**Figure 1:**
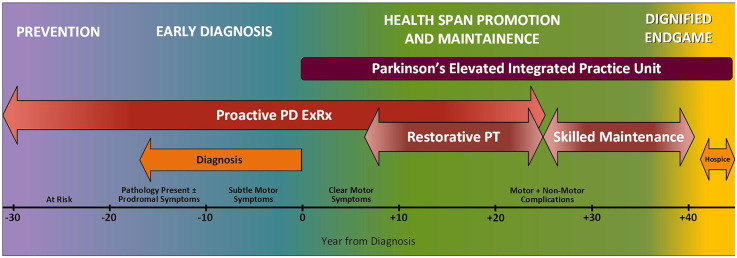
Ideal journey of a person with Parkinson’s disease. The ideal journey will accelerate diagnosis, extend the healthspan, and make the endgame shorter and more meaningful.

After exploring problems with the current state of care, our PFAC helped us map the ideal journey (Figure 1), which we then separated into four main stages. Starting at the very earliest point, when someone is at risk for PD but has no pathology, they need **Prevention** or delay of disease. Once someone has clinically relevant symptoms such as rapid eye movement sleep behavior disorder (16), they need a rapid **Early Diagnosis** and referral to a Parkinson's IPU. There have been several very promising advances in how to detect Parkinson's earlier and with greater certainty. They include: (1) CSF biomarkers (17) (2) alpha synuclein skin biopsies (18) and (3) advanced brain imaging techniques and artificial intelligence (19). The earlier a person is diagnosed, and the earlier a person receives comprehensive healthcare, the better the prognosis. Diagnosing PD early is important because it allows initiation of disease modifying treatment as early as possible and reduces unnecessary suffering, healthcare utilization and cost.

The remaining phases of a person's journey take place within the IPU: first **Healthspan Promotion and Maintenance** (the longest phase of the journey spanning several decades and including different forms of rehabilitation) and finally, the **Dignified Endgame** in which hospice care is provided as part of the IPU. We take the term ‘Endgame’ from Samuel Beckett's drama of the same name (20). Our PFAC members expressed appreciation for the use of this term because it clearly describes a phase they know is coming and should be approached with care and compassion. Such candid acknowledgment facilitates preparation for and control of their own end-of-life plans.

For the rest of this paper, the focus is exclusively on the care that unfolds within the IPU (**Healthspan Promotion and Maintenance** and **Dignified Endgame**)– the vehicle through which we propose to provide financially sustainable proactive integrated health care.

## 4 The integrated practice unit

First described in 2013 by Porter and Lee, an IPU provides care organized around a medical condition or set of closely related conditions that is delivered by a dedicated and connected multidisciplinary team who assume joint accountability for quality and cost of care (3, 4). In the *Parkinson's Elevated* IPU, we will provide integrated proactive and reactive care. Strategic, insight-driven proactive care will reduce the likelihood of needing complex, expensive reactive care. Outcomes that matter to the patient will serve as our barometer of clinical success. Existing outcome measure sets are compared elsewhere (21) but, in brief, the *Parkinson's Elevated* IPU will deploy fidelity measures (eg: percent of patients adhering to the PD Exercise Prescription) along with measures of quality of life such as those proposed by the International Consortium for Health Outcomes Measurement (22).

New models of care must focus on cost because the current healthcare system cost structure is not aligned to incentivize best patient care, nor is it fiscally sustainable in the long-term. The current fee-for-service (FFS) healthcare payment model supports only reactive care by paying for each service or procedure rendered individually to a patient with disease or disability. In the FFS model there are no financial incentives to provide proactive or integrated care, or care that achieves outcomes that matter to the patient. We describe in the final section of this perspective how an IPU properly aligns financial incentives through joint accountability between insurance companies and healthcare providers to ensure high quality care at a sustainable cost. Because Parkinson's Disease affects every organ system in the body, it is an ideal condition around which to pilot an IPU (1). The approach we present will reduce both disease and financial burden over the lifespan of the person with PD while simultaneously improving their quality of care and patient experience.

## 5 The five elements of the *Parkinson’s Elevated* integrated practice unit

The general tenets of an IPU are described in a “Playbook for Health Care Leaders” (4). Guided by this, we have defined 5 core clinical elements of the *Parkinson's Elevated* IPU.

### 5.1 Integrated, interdisciplinary care, including primary care

The backbone of the IPU will be integrated care that is “coordinated across professions, facilities, and support systems, continuous over time and between visits, and tailored to patient and family needs, values, and preferences” (23). To achieve this as well as to maintain control over total cost of care, a person's primary care physician (PCP) and movement neurologist must work closely, as originally proposed by Bloem, Okun, and Klein (24). But what does this look like in practice? We are currently running a healthcare delivery experiment by integrating a primary care physician (TWS) into the Intermountain movement disorders clinic to see only PwP and their carers. To date this has been met with immense satisfaction from patients and physicians. Care coordinators (Nurse, Exercise Health Coach) provide support for this integrated approach (6).

### 5.2 Proactive implementation of the Parkinson’s exercise prescription (PD ExRx)

One major goal of the *Parkinson's Elevated* IPU is to promote early and high adherence of PwP to the Parkinson's Exercise Prescription (PD ExRx) which we have detailed elsewhere (25). In short, when we refer to PD ExRx, we are referring to an exercise prescription that includes four components. The first component, PD ExRx _aerobic exercise_, has the most evidence to suggest positive disease modification (26–34). The other three components of the exercise prescription—PD ExRx _resistance training_, PD ExRx _flexibility training_, and PD ExRx _neuromotor training_—have not been shown to be directly disease modifying; however they can improve physical function (35) and are associated with better long-term motor outcomes (36). Long-term participation in these modalities can also prevent frailty and debility which if left unaddressed significantly increase morbidity and mortality (37, 38). The neuromotor component of the prescription is particularly important for improving locomotion, improving balance, and reducing falls (39).

Although the IPU will ultimately focus on implementation of all four components of the PD ExRx, we have focused our initial efforts heavily on PD ExRx _aerobic exercise_ because a cure for PD remains elusive (40), and decades of drug trials have failed to produce a disease-modifying treatment (41, 42). Medication and surgery provide a way to mitigate disability and return some quality of life, but they do not slow disease progression. A growing body of evidence suggests that aerobic exercise may be disease-modifying when performed at high intensity (30 min three times a week at 80%–85% heart rate maximum) (26–29). Although the exact mechanism for probable disease modification is unknown, one reason why aerobic exercise is so beneficial for people with PD is that it causes positive health related benefits on the endocrine system, the inflammatory system, and also the neurotrophic system (Luthra et al. manuscript accepted pending revision). If the PD ExRx _aerobic_ can be deployed at the earliest phases of the disease, longer-term complications will likely be delayed: the very definition of proactive care. Although there are many areas of care that can be provided to PwP proactively as detailed in the perspective by Bloem et al. (1), here, we highlight aerobic exercise specifically, because failure to routinely deploy a treatment that most likely slows disease progression is a huge missed opportunity in current PD care.

**Figure 2:**
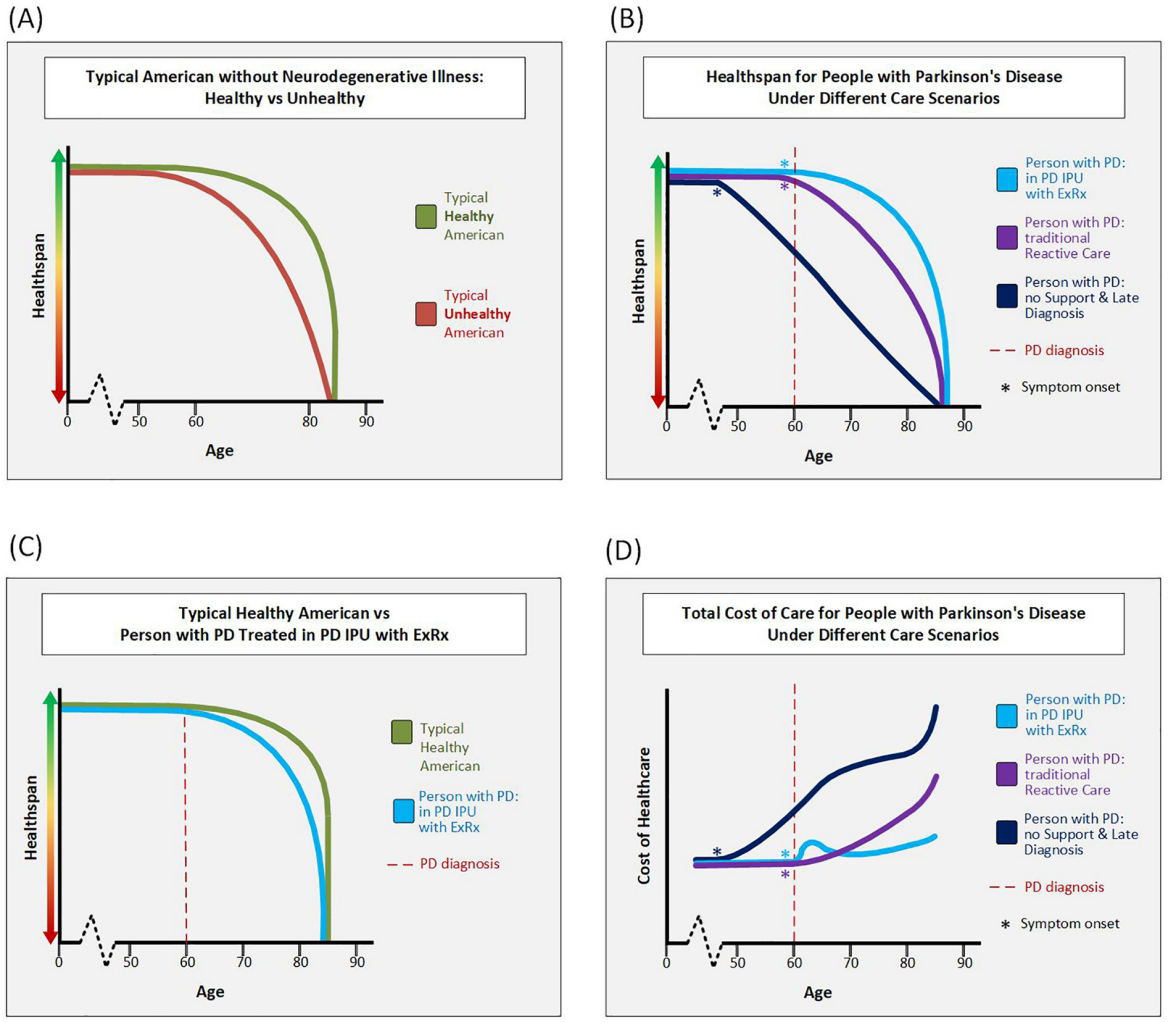
Hypothetical clinical and cost trajectories. **(A)** This figure depicts the hypothetical healthspan of typical Americans without PD or other neurodegenerative illness. A typical unhealthy American does little to promote health and may develop metabolic syndrome which increases risk of type II diabetes, heart disease, and/or stroke. These unhealthy Americans likely experience a “marginal decade” (75) or more at the end of their life in which they are alive, but have poor health and poor quality of life. In contrast, by early middle age at the latest, a typical healthy American engages in multi-modal exercise, healthy eating, quality-sleep, and care for their mental health. As long as these healthy Americans do not develop a disease out of their control such as cancer or a neurodegenerative illness, they maintain a high healthspan until their time of death. **(B)** This figure depicts the hypothetical healthspan of 3 PwP all diagnosed at age 60, but treated under different care scenarios. The person with PD treated in the *Parkinson's Elevated* IPU with the PD ExRx maintains the highest healthspan. The person with PD who receives good neurologic and family support, but is treated in the traditional reactive care model has more disability in their later decades than the person in the IPU. The third person with PD is someone without support who is diagnosed over a decade into their decline and thus starts at a much lower level of health at time of diagnosis. This person continues a precipitous decline due to continued lack of support. **(C)** This figure overlays the hypothetical typical healthy American and the hypothetical person with PD treated in the *Parkinson's Elevated* IPU model. While the healthspan of the person with PD is lower than that of the healthy person without PD, it is not that much lower. **(D)** This figure compares the hypothetical total cost of healthcare for PwP in different care scenarios. The lifetime cost is denoted by the area under each curve. Note that for a person with PD with late diagnosis and little support the cost of care is hypothesized to rise even before diagnosis. For a person with PD in the *Parkinson's Elevated* IPU, cost is hypothesized to rise at diagnosis when resources are invested in proactive care, but over time the cost curve falls below that of a person with PD treated in the traditional reactive-only care model. We anticipate that the *Parkinson's Elevated* IPU will reduce long-term costs through steering patients toward a more benign trajectory for both their PD and non-PD comorbidities (because exercise and care coordination help far more than just PD). In a more benign trajectory, less reactive care is needed. When reactive care is needed however, there will be reduced spend by bringing most of the care into the unit. Finally, transition to hospice at the right time will save high-but-futile spending in the last few months of life. Note that the IPU curve is the only one without steep rise in cost at end of life due to the team being able to proactively transition PwP to hospice. Abbreviations: PD, Parkinson's disease; PwP, people with Parkinson's disease; IPU, integrated practice unit.

Figures 2A–C detail the hypothetical trajectory of healthspan for people with and without Parkinson's and demonstrate that healthspan is modifiable based on interventions such as the PD ExRx started in mid-life.

Despite mounting evidence that the PD ExRx will meaningfully improve healthspan (29), many PwP are not routinely engaging in exercise for a variety of well-studied reasons (43–45). Certainly, the American healthcare system does not facilitate exercise adherence because financial incentives are not aligned with delivery of such a proactive intervention. In construction of our *Parkinson's Elevated* IPU, we are using an implementation science-informed approach to expedite the availability of this intervention to patients. So far, we have identified five key barriers to implementing the high-intensity aerobic exercise component of the Parkinson's exercise prescription (PD ExRx_aerobic_) at Intermountain Health (Table 1). We are piloting solutions in a single-site 48-person feasibility/efficacy/cost effectiveness study.

**Table 1:** Barriers and solutions to implementation of the Parkinson's disease exercise prescription (PD ExRx).

Barrier	Solution
Misaligned financial incentives	We are working with our integrated payer Select Health (DMH) to build a system in which the care team is jointly accountable for quality and costs of care. This will allow freedom to direct resources toward exercise.
Lack of physical therapist training to support PwP in achieving the PD ExRx	The high heart rate intensity intervention is led by a seasoned neurologic physical therapist (SAO) to develop a PT-based pathway to deliver the PD ExRx at Intermountain. A neurologic physical therapist with implementation research expertise and extensive experience in delivering and scaling evidence-based PT for PD (MRR) is overseeing pathway development.
Lack of clinical access to maximum CPET which is needed to determine maximum heart rate*	The protocol for CPET testing from prior PD-specific clinical trials (26, 46) is being used and overseen by experienced PD exercise physiology researchers (DMC, GJG). Full details are provided in (47).
No existing system to coordinate care between neurologist, physical therapist, CPET	A project coordinator (MMKB) completes all patient scheduling for the PD ExRx components. We will track the cost and resource utilization of this role to allow future scaling.
No existing mechanism for frequent-contact coaching—which is what has been used in successful clinical trials of exercise for PwP (26, 46).	The same project coordinator (MMKB) serves as an exercise coach. She has a certification as a health coach and has received training from experienced exercise interventionalists (DMC, GJG). Analysis of the time and training for this role will allow future scaling.

We are piloting these solutions in a single-site 48-person feasibility study. In this study, a neurologic physical therapist (SAO) screens participants. If the participant is not suitable for the PD ExRx intervention, they receive restorative physical therapy (further described below) and may be eligible for the intervention in the future. If the participant is currently suitable for the ExRx intervention, they undergo a cardiopulmonary exercise test (CPET) performed by an exercise physiologist trained in PD (MNF). The neurologic physical therapist then gives the participant their aerobic exercise prescription based on maximum heart rate obtained during CPET. The prescription is to achieve 3 sessions per week of 30 min of aerobic exercise at 80%–85% heart rate max (plus warm up/cool down). The neurologic physical therapist supervises participant exercise using in-person sessions until the person with PD demonstrates ability to use the Polar heart rate monitor (provided to them as part of the study) to measure heart rate and consistently hit their individually tailored target heart rate. The project coordinator (MMKB) extracts and monitors participant heart rate data and serves as their coach via weekly contact to ensure they continue to meet their target. At the discretion of the physical therapist the other three components of the full PD ExRx (resistance training, flexibility, neuromotor) are prescribed for the participant.

*To write an accurate ExRx, accurate maximum heart rate is needed. The most rigorous method to determine maximum heart rate is a maximum CPET (48). This is how the exercise prescriptions have been set in clinical trials (26–28, 46). Common formulas to estimate maximum heart rate do not work for many PwP because they have autonomic dysfunction or are on chronotropic medications (49, 50). It is unknown if an accurate maximum heart rate can be determined in some PwP without CPET. Abbreviations: CPET, cardiopulmonary exercise test; PD, Parkinson's disease; PT, physical therapy; PD ExRx, Parkinson's disease exercise prescription.

### 5.3 Specialist network for restorative and skilled maintenance therapy

Although we will always try to enroll PwP early in their disease course into all four-components of the PD ExRx, some people may not be ready for the high-intensity aerobic exercise component initially, and others may never be able to undertake such rigorous exercise because they enter the IPU late in their disease course or have non-modifiable comorbidities preventing participation. Additionally, although implementing the PD ExRx early should reduce disability, none of the treatments we deploy are curative; many people will still experience functional decline over time and will require adaptations to the ExRx. At the first sign that a person with PD could benefit from person- specific adaptations to therapy, they must be referred early and often to specialized physical therapy—which along with continued exercise and physical activity will definitively improve PD motor and non-motor symptoms and physical function (25, 29, 35, 36, 38, 51–59). When needed, PwP should also be referred to specialized occupational and speech therapy (58). This therapy should be conceptualized in two phases: restorative therapy and skilled maintenance therapy (See Figure 1).

It is important to stress that not all people with PD will respond to therapy in the same way. This is why people with PD should be treated by neuroPTs who have the skillset to prescribe appropriate person-specific therapy based on how an individual responds. It is also the case that many people with PD have a variety of comorbidities such as mobility/osteoarthritis or cardiovascular issues and cannot perform aerobic exercises at moderate to high intensity. These individuals will receive individualized exercise prescriptions.

*Restorative therapy* is focused on fixing or mitigating a deficit to restore function. For example: PT to improve balance, swallow therapy by a speech and language pathologist to reduce aspiration, and occupational therapy to improve hand dexterity for dressing or handwriting. Most existing physical, occupational, and speech/swallow therapy is set up as restorative therapy in which the therapist works with the patient to improve function or fix a specific deficit. However, many PwP can also benefit from *skilled maintenance therapy* toward the end of their life to maintain basic physical function– e.g.,: safe mobility in the home, communication, and basic activities of daily living (60). Healthspan at this late stage has clearly fallen from the pereson's prior baseline level and may not be able to be restored. However, with skilled maintenance therapy, further decline may be forestalled and some measure of health preserved to allow a higher quality last phase of life.

There are three problems with existing therapy infrastructure that limit high-quality restorative and skilled maintenance therapy. The first is that a fragmented care system does not even direct PwP to PT in appropriate numbers. In 2016–2018, only 18%–25% of participants in the Parkinson's Foundation Quality Initiative were referred to PT (13). To solve this problem PwP treated in our IPU will be referred to PT through an integrated pathway. The second problem is that, even when PwP make it to PT, they often do not receive specialized Parkinson's PT (61, 62). Although it is recommended that PwP be treated by rehabilitation therapists specialized in PD (63–65), patients frequently cannot access these specialized clinicians due to transportation barriers or lack of appropriate referrals. To solve this problem, a network modeled after Parkinson Net (66) is needed. In this Dutch network, PwP are preferentially directed to therapists who have received extra training and maintain a certain minimum volume of PwP as patients. Parkinson Net has demonstrated improved quality and reduced costs for PwP (67). This approach is becoming more recognized and available in the United States, with examples in North Carolina (68) and in California (69).

The third problem with the existing therapy infrastructure is lack of insurance funding for rehabilitation focused on “maintenance.” Many FFS insurance providers, including Medicare, base authorization and payment on the short-term achievement of functional gains rather than goals to ‘maintain’ function over the long-term. This forces therapists to discharge patients once no further ‘rehabilitation’ is possible, when in fact patients would still benefit tremendously from skilled therapists helping to maintain their function. In skilled maintenance, therapy progress should be measured by absence of decline over longer-term episodes of care.

### 5.4 Physician specialist care provided in the unit and through a tight network of subspecialists

All people—but especially PwP in whom every organ system is affected —need clinicians who understand the whole person and orient care around them (24). Wherever possible the *Parkinson's Elevated* IPU will bring the first several steps of organ-specific care into the unit. When people's needs exceed what can be provided directly by the IPU, referrals will go to specific physicians within a tight referral network. This will promote higher quality and more coordinated care because the specialists will develop PD-specific expertise and the IPU team need only coordinate with 1–2 specialists per organ-system.

### 5.5 A dignified endgame: hospice provided in the IPU

A **Dignified Endgame** involves a smooth and supported transition to hospice in which the care team that has followed the person with PD through their **Health Promotion and Maintenance** phase continues to be involved in their care. This continued involvement preserves a multi-year relationship in addition to facilitating the ongoing technical management of a disease that renders many palliative pharmacologic agents contraindicated. It also promotes a *proactive* transition to hospice because the patient knows they will continue with the care team they trust. Such an arrangement is not possible in a FFS system: in that system, once a patient enrolls in hospice, insurance will no longer pay for them to see any clinicians outside the hospice team. Many of our patients have cited this as the main reason they do not wish to enroll in hospice at all. We are piloting a program with Intermountain hospice in which the movement neurologist serves as attending physician when a patient transitions to hospice. To-date this partnership has been met with high patient and family satisfaction as well as ongoing support from Intermountain Hospice leadership.

## 6 How will we pay for this: joint accountability for quality and cost of care

Programs that provide proactive integrated care likely produce the best health outcomes; yet existing financial incentives are not aligned to support these programs (70). Rather, in our current FFS system, care is provided in medical departments only after the problem has occurred. Because such reactive care is the only thing insurance will pay for, it has become the only type of care clinicians are set up to provide. Care providers financially benefit when the number of billable services are maximized (71). Insurance companies financially benefit by denying services or delaying care. When care of a person is reduced to discrete reactive care snapshots like this, patients suffer while care providers and insurance companies fight over the “necessary” and “clinically justifiable” dollars and cents of reactive care.

The *Parkinson's Elevated* IPU will solve these problems through a value-based care model (72) in which financial incentives are aligned with clinical best practices including proactive care. Specifically, we are planning a capitated (risk-sharing) model in which the insurance company and healthcare provider in close conjunction with the clinical care team jointly assume responsibility for quality and cost of care for all patients in the IPU. In such a model, the IPU will be paid a set amount of reimbursement per patient per year. The IPU will then be responsible for using that money to effectively manage all the care (not just Parkinson's specific care) for all the patients attributed to the IPU. If the average cost of care falls below the annual set amount, cost savings will be shared by both healthcare provider and insurance company. (Note—to prevent unplanned catastrophically high costs from crippling the program, “stop-loss” insurance will be in place.) In such an arrangement, because we will be able to re-direct funds towards services not traditionally covered in a FFS system, we will be able to implement evidence-based mechanisms of care such as the proactive PD ExRx. Success will be measured not by volume of billable services rendered but rather by outcomes that matter to the patient weighed against total cost of care. Figure 2D demonstrates the theoretical life-time cost of care for PwP in different care scenarios.

## 7 Conclusion

The ideal journey of a PwP is one of early, compassionate and optimistic diagnosis followed by referral to a Parkinson's Disease Integrated Practice Unit. Our proposal for the *Parkinson's Elevated* IPU at Intermountain Health uses integrated care to deliver a heavy dose of sustainable proactive care while still providing any reactive care needed. The five core elements of the *Parkinson's Elevated* IPU are: 1- Integrated, Interdisciplinary Care, including Primary Care, 2-Proactive Implementation of the Parkinson's Disease Exercise Prescription (PD ExRx), 3-Specialist Network for Restorative and Skilled Maintenance Therapy, 4-Physician Specialist Care Provided in the Unit and through a Tight Network of Subspecialists, and 5- A Dignified Endgame: Hospice Provided in the IPU. To ensure financial sustainability, the healthcare providers in the IPU will be jointly accountable with insurance companies for the quality and total cost of care delivered to PwP in the IPU.

As the national debt continues to increase (currently $35.91 Tr in late 2024) (73), the age of the population continues to increase, and healthcare expenditures continue to increase ($4.8 Tr in 2023) (74), there has never been a more urgent time to improve the quality and decrease the cost of healthcare in the US. The *Parkinson's Elevated* IPU has the potential to greatly improve the healthspan of PwP while reducing the overall cost of care over the course of the disease. Creation of objective healthspan indices as well as the ability to measure cost over a lifetime in people with and without PD as they age with and without proactive measures are needed to prove the value of this conceptualization of treating PwP. However, there is enough circumstantial evidence supporting the hypothetical healthspan and cost curve trajectories we have proposed (Figure 2), that we are planning to implement the *Parkinson's Elevated* IPU now, rather than wait for more data.

## Data Availability

The original contributions presented in the study are included in the article/Supplementary Material, further inquiries can be directed to the corresponding author.
